# A qualitative study to assess community barriers to malaria mass drug administration trials in the Gambia

**DOI:** 10.1186/1475-2875-13-47

**Published:** 2014-02-04

**Authors:** Natalie J Dial, Serign J Ceesay, Roly D Gosling, Umberto D’Alessandro, Kimberly A Baltzell

**Affiliations:** 1 Department of Global Health Sciences, University of California San Francisco, 50 Beale Street Ste. 1200, San Francisco, CA 94105, USA; 2 Medical Research Council Unit, The Gambia, Atlantic Road, Fajara, P.O. Box 273, Banjul, The Gambia; 3 University of California San Francisco, Departments of Global Health Sciences and Family Health Care Nursing, 2 Koret Way, N431-M, San Francisco, CA 94143, USA

**Keywords:** Malaria, Mass drug administration, Compliance, Adherence, Qualitative interviewing

## Abstract

**Background:**

Mass drug administration (MDA) is a strategy widely used in the control of human parasitic diseases but has been rarely attempted with malaria, the most common and dangerous parasitic disease in humans. MDA is an intervention strategy that involves simultaneously dispensing treatment to an entire population in a given geographic area. With some areas in sub-Saharan Africa documenting a decline in malaria transmission, the feasibility of MDA to further reduce malaria transmission is being considered. Understanding community perceptions of such an activity is vitally important for the design of the study and gaining the support of participants in order to maximize compliance and adherence.

**Methods:**

A qualitative study to assess factors likely to influence community acceptance of MDA in the seasonal and low malaria transmission setting of The Gambia was conducted. Using in-depth interviews, the perceptions, knowledge and attitudes of medical personnel and community members who have undergone MDA trials in The Gambia were investigated.

**Results:**

Several major themes emerged, namely: 1) the importance of timing of rounds of MDA doses for maximum participation; 2) the need to educate the target population with accurate information on the procedures, drug regimen, and possible side effects to enhance adherence; 3) the need for continuous sensitization meetings to maintain and increase uptake of MDA; and, 4) the importance for defining roles in the delivery and assessment of MDA, including existing healthcare structures.

**Discussion:**

To increase the likelihood of participation in MDA trials in this setting, activities should be undertaken just before and during the rainy season when community members are less mobile. Importantly, fears regarding blood sampling and side effects of the drug regimen need to be addressed prior to the start of the trial and repeated throughout the study period. Accurate and frequent communication is essential, and village leaders should consistently be included in sensitization meetings to enhance community participation. Additionally, village healthcare workers should be included in training and implementation, with supervision by a fieldworker permanently posted in every few villages during the trial. Future collaboration with Senegal may prove important for enhanced elimination efforts in The Gambia.

## Background

### Malaria control and elimination

Of the areas of the world where malaria remains endemic, approximately one-third of the countries are pursuing strategies to transition from control to elimination of the disease [1]. Transmitted by the female *Anopheles* mosquito, malaria still infects approximately 200 million people per year and accounts for 600,000 deaths annually [2]. The majority of the disease burden afflicts sub-Saharan Africa, where the most deadly of the parasites, *Plasmodium falciparum,* is most prevalent. Recent findings note that in low endemic countries and seasonal settings, a large proportion of *P. falciparum* infections are carried asymptomatically and in low densities, resulting in false negatives using normal diagnostic tests such as blood slide microscopy or malaria rapid diagnostic tests [3]. Treating these asymptomatic infections is likely to have a greater effect on malaria transmission than just treating symptomatic patients who report to health facilities [4].

### Elimination efforts

Mass drug administration (MDA) is the simultaneous dispensing of treatment to an entire population in a specific geographic area, regardless of presence or absence of disease symptoms and without diagnostic testing. MDA is the control strategy of choice in other parasitic diseases, such as lymphatic filariasis, schistosomiasis, and onchocerciasis [5-7]. However, current studies of MDA for malaria have shown mixed results, with some evidence of success yet no long lasting effects in either sub-Saharan Africa or the Asia Pacific [8-12].

Specific to malaria infection, there is emerging evidence that MDA may be more effective when targeting both the asexual and the sexual (gametocyte) stages of the parasite’s lifecycle [4]. Although insufficient alone for malaria elimination, destroying both the asexual stages and the gametocytes in human hosts can have a significant effect on reducing transmission if MDA is successfully implemented before transmission season begins [13,14]. Such evidence has provided improvements to trial designs, but evaluation of implementation methods is also needed to more comprehensively improve MDA programmes.

### Identifying barriers

One notable cause of MDA programme failures is the lack of coverage of the target population and participant adherence to the drug regimen [15]. It is estimated through modelling that coverage of the target population in excess of 80% is needed for MDA to be successful. This assumes a static population and does not account for humans migrating into the MDA area with new infections [15].

Research indicates that misconceptions within MDA target populations contribute to lack of initial compliance and ongoing adherence to the drug regimen, and therefore are barriers to success [16,17]. Specific misconceptions attributed to those implementing MDA campaigns include lack of trial justification to the community, community misunderstanding of the medicine being administered, who are at risk for the disease, and the need to target asymptomatic carriers [18]. There are limited reports focusing on how best to change MDA campaigns to increase success within the community. These studies do, however, suggest that a large part of successful MDA implementation is due to community education and health worker training [18,19].

### Malaria mass drug administration in the Gambia

In 1999, an MDA for malaria was trialed in the seasonal and low-transmission setting of the Gambia, and did not yield a significant reduction in transmission [12]. In addition, a 2001 follow-up study exploring the shortcomings of this malaria MDA trial had narrow participant inclusion, which limited its ability to provide useful information for improving future MDA trials [20]. Further understanding of the barriers to adherence and community mobility has the possibility of enhancing the effectiveness of MDA trials.

Importantly, investigations into the lack of adherence in studies of MDA should involve a spectrum of stakeholders and participants, such as health care workers, village leaders, government officials and community members. Therefore, this qualitative study was conducted to explore the community barriers to MDA trials by exploring the perceptions, knowledge and attitudes of participants in a malaria MDA trial in The Gambia that took place in 1999. The goal of this study was to identify barriers and facilitators to the MDA trial with the goal of increasing adherence in future campaigns.

## Methods

### Study population and study sites

This study predominantly targeted individuals affiliated with a malaria MDA trial from 1999 in The Gambia, but also included several individuals involved in a 2010 trachoma MDA trial in The Gambia. Participants were selected from various levels of involvement in the MDA trials. This included researchers, fieldworkers, government officials, and healthcare personnel. Additionally, the study included village members from rural communities within the North Bank Division of The Gambia who participated in the 1999 malaria MDA trial.

### Procedures

Participant recruitment was carried out through a mixed-sampling approach, including both purposive and snowball sampling techniques. Key informants were identified in collaboration with the Medical Research Council Gambia Unit, the National Malaria Control Programme (NMCP), the Centre for Innovation Against Malaria (CIAM), and various major urban health facilities. Key informants were contacted to participate via email or phone. Initial participants were drawn from positions considered as “stakeholders” in previous campaigns [12], such as the researchers, project managers, fieldworkers and government officials. Stakeholders were asked to both participate in the interview and to refer individuals to participate in the study. Likewise, village leaders of participating districts were first approached, and asked to engage in the interviews and then recruit members of their community to participate. Only individuals who did not wish to participate were excluded.

### Data collection

A pre-piloted, semi-structured interview questionnaire was used for data acquisition. The research constructs aimed to address: 1) beliefs of key determinants to successful MDA trials; 2) reasoning behind non-compliance and non-adherence; 3) communication between acting parties; and, 4) how the trachoma and malaria MDA trials compared and contrasted. Interviews were audio-recorded and transcribed at the end of each day with the assistance of a fieldwork translator.

### Data analysis

Interviews were transcribed using an inductive, iterative process including: 1) familiarization with individuals, questionnaire, and study area; 2) coding the data by abstracting texts; 3) identifying themes and concepts; 4) refining; and, 5) report writing. All analysis of interviews was done by hand coding, and emerging themes were identified based on frequency of appearance. Discrepancies in coding were reviewed by a fieldwork assistant and further reviewed by the fieldwork mentor when necessary.

### Ethical considerations

The Medical Research Council The Gambia’s Institutional Review Board and the University of California, San Francisco’s Committee on Human Research approved this study. A written informed consent form was read and signed by all participants.

## Results

Interviews were conducted with 26 key informants, located in the towns of Fajara, Basse, and Farafenni, and in the villages of Bambally, Kunjata, Sarakunda, Pallen fulla, and Kanikunda Suba. Key informants included a variety of stakeholders and community members involved in the 1999 malaria MDA trial in The Gambia, as well as several involved in a 2010 trachoma MDA in The Gambia (see Table 1 for key informant abbreviations).

**Table 1:** List of key informants by strata, involved in malaria mass drug administration unless otherwise specified

**Strata**	**Abbreviation**	**Number of key informants**
Researchers	R	3
Fieldworkers	FW	2
Government officials	GO	3
Village reporters	VR	3
Village healthcare workers	VHCW	3
Community members	C	6
Opinion leaders	OL	4
Trachoma researchers	TR	2

The following major themes were identified:

Theme 1 Participants emphasized that timing of the mass drug administration was essential for a successful campaign, but opinions differed on whether administration during the dry or rainy season would improve participation

Many village residents believed MDA campaigns should occur during the dry season, due to farming obligations in the rainy season.

The best time is during the dry season, because during the rainy season everybody has gone to the farms.

(VHCW 3)

It should be in the dry season, because during the rainy season people are busy.

(C 5)

It can be given during the dry season, because I remember during the rainy season I took the medication and felt dizzy and could not work.

(C 6)

Conversely, researchers and opinion leaders argued that community members use their time off in the dry season to travel, therefore participation would be maximized during the rainy season.

The best time to do a trial is during the rain, from June onwards. Because everyone sees themselves staying in the village, in the community doing their fun activities, they don’t move out. But during December, dry season, people travel anywhere they like to. It is very difficult to control them during the dry season.

(R 3)

The medication should be given in the rainy season. The only disadvantage with that is that if the medication is if they feel dizzy they cannot work in the farms.

(OL 4)

I would prefer it in June because June is beginning of rainy season in The Gambia. It is a month that people are busy, but it is a month that is good for such to happen.

(R 2)

Absence from the village during time of administration was identified as an obstacle to MDA success. Travelling outside of the village often extends across the border to Senegal. Several government officials suggested that Senegal be included in a future MDA trial.

*At least 20*% *of your population floats. So I think that’s the major problem—trying to catch people who aren’t there and know that you haven’t caught them, even. I think it’s a problem because in The Gambia it’s surrounded so much by Senegal. The borders, of course, are extremely porous as they should be—the countries are at peace, people should be able to move around.*

(R 1)

Because you know there’s cross-border contamination. You will not be able to achieve real impact with MDA without collaborating with Senegal.

(GO 3)

Theme 2 Adherence and compliance to the MDA was dependent on participants’ understanding of the procedures, drug regimen, possible side effects, and eligibility for the trials

A concept frequently reported by fieldworkers and villagers was the fear of blood samples being sold. Villagers were familiar with newer methods of malaria diagnostics that only require a finger prick for detection of parasitemia, therefore skepticism increased with the requirement of blood taken from the arm. Researchers and fieldworkers reported an understanding of the need to bleed participants from the arm, but villagers themselves did not express this understanding.

They didn’t participate because of the blood people were taking. They said eventually they came to believe that they would sell the blood, they would sell our blood. This led to a lot of refusals.

(VR 2)

Well sometimes bleeding is a problem to these people, like frequent bleeding. Some people might think it will only go for a few months and stop, so when it’s continuing some people may change their minds.

(R 3)

*The reason why some of them denied is because they know with malaria, you can tell just by taking the blood here* [points to finger tip] *but then they started to take blood from here* [points to arm] *they said it was too much to test for malaria.*

(VR 2)

Fieldworkers and community members struggled with compliance and adherence due to the dosage including too many pills, particularly with the frequency of administrations.

A participant who said ‘I want to take part, but these drug that you are giving to us is too much’. It is seven tabs, and he said ‘this is too much, I cannot swallow all this’

(FW 1)

They just refused and said it was too many tablets and they did not want to drink that many tablets.

(OL 4)

Villagers reported physical side effects of the medicine, which reduced their adherence to the MDA drug regimen. The side effects bothered them most during the rainy season because it stalled their work in the farms, therefore interfering with the daily provision to their families.

We drank the medication, but when we went to the bush to do the farming, we had to lay down and sleep because it made us dizzy. It was not easy with our stomachs.

(C 3)

When they drank the medication they get dizzy, and when they go to the farms they cannot do anything.

(OL 1)

Some refusals were reported based on a state of pregnancy or intention of pregnancy. Some women of childbearing age chose to not take the medication for this reason.

I feel that the reason most didn’t drink the medication is because they told us if we are just a few months pregnant we cannot take the medicine.

(C 4 and 5)

Theme 3 Sensitization sessions, the formal educational meetings that occur between researchers and study population, were reported as the most important component of an MDA trial

Key informants consistently reported that the most important element of a mass drug administration is the sensitization of the community to the study and the procedures.

It is sensitization, it has to be very clear to them and it has to be before the study starts. If there is any procedures, blood taken, whatever amount, you have to make sure that during the sensitization everything is solved.

(R 2)

I believe if there is proper sensitization, people will understand.

(OL 2)

I didn’t have enough time to disseminate the information to the villages. Because prior to the start of the trial we said we wanted to go around to the villages, but it did not materialize.

(FW 2)

Researchers and opinion leaders reported the influence of village members who often diagnose and treat diseases without a legitimate healthcare certification. Key informants recognized that these people may distort the trial results in some way, and sensitization efforts specific to them should be made.

Sometimes you have the shopkeepers giving the drugs, the antimalarial drugs.

(R 3)

Sensitization should be very wide and extend to the shopkeepers, drug sellers, and whatever else in the community, so they do not interfere with the study.

(OL 3)

A new element of the sensitization campaigns that was suggested consistently across key informants was use of the media. Particularly in comparing the trachoma and malaria MDA trials of The Gambia, it became apparent that the media was not used in the 1999 malaria MDA campaign.

Sensitization, we used various media for that, what with the electronic and billboard structures.

(TR 1)

It starts with various education channels to communicate with the community. They use the media, like radio for example. They develop a message in the various languages and they use those to relate. The radio is not available to all, but it is available to many.

(GO 1)

We had two radio stations before the distribution, where we sensitized the individual villages and tell them the effect of trachoma. And we allow phone calls even, to call in with any of their questions and we answer it. So it was effective.

(TR 2)

Over time, the attitude toward intervention trials in the rural communities has transitioned from skeptical to more accepting.

If you can look at the time that the MDA was done, there was not a lot of awareness. There is more awareness now, and those who are part of the programme are much stronger now so they can convince those people who are not ready to be part of the programme.

(C 1)

I know things are changing from 1999 to now, because now people are conferring consent to research in The Gambia because sensitization is an ongoing thing.

(OL 3)

Initially, there was no awareness. But since the campaign started there was awareness, people were sensitized, and people became to know the importance of the project.

(VHCW 2)

Sensitization sessions themselves should be based on community preferences.

Involve us; we should be the example.

(C 6)

Normally we don’t suggest, we go to the community and ask what day would be best for them.

(R 2)

This is why fieldworkers have their own time to visit their study subjects: what time is convenient? As you stay with them in the community, you will know what time is convenient. It all depends on how you’re going to plan your work according to their schedules

(R 3)

Theme 4 Those involved in MDA trials need defined roles and improved cohesion. Existing healthcare structures must be taken advantage of to maximize efficiency and effectiveness of information dissemination, compliance, and adherence

Villagers, fieldworkers, and researchers emphasized the need for improved communication between and among themselves.

It should be communication between the ones that are the researchers--the people who are bringing this type of programme into the community--and the community leaders.

(VR 3)

Such a programme needs a lot of dialogue between the people and the person disseminating the information and the person implementing the programme. You need to know the community very well—you need to know which individuals are barriers to the campaign, and be able to approach them before the project begins.

(FW 2)

Communication between all participants must be truthful, consistent and frequent. This includes an initiative on all parts to identify and absolve barriers to trials.

You have to be sure your staff is telling you really what’s happening, because you can’t go to everyone yourself.

(R 1)

I feel there is no good interpreter between the doctors and the local community. And sometimes this delays jobs being done.

(OL 2)

There is not a consensus amongst key informants whether the results of the 1999 MDA were reported back to the target populations.

No we did not go to them to tell them the programme had failed. They should know, they need to know so at the end of the day we will be free from blame.

(FW 2)

In regards to the trial-- I was a part of it, but afterwards there was no feedback for me. I believe this is something that is lacking from my own point of view; giving feedback to the community, it’s not done.

(R 2)

Several key informants suggested a fieldworker, closely related to the researchers conducting the trial, be permanently posted in every village, or every few villages.

There needs to be a type of person that can be relied upon by the general community for questions.

(VR 1)

Some of them come before even and start asking questions. Because even out of working hours, some people can be passing by and come into my house and say ‘I have a doubt’.

(FW 1)

According to many key informants, local staff must be used more effectively. Although this will require additional training efforts, it is reported that involving village health care workers and local staff will engage communities more quickly and effectively.

I recommend that if you want to do this kind of thing, you link with the primary health care worker in the village. If you link with that man, you have a lot of influence over the people.

(VR 2)

If they are taking from our practice, they should be taking the existing structures from the community. And that’s what we did; we used the existing structures with emphasis on training.

(TR 1)

Opportunities to recruit traditional healers should be taken advantage of. Key informants from the trachoma MDA trials explained participation of village health workers called Nyaterros, or ‘friends of the eye’. These individuals proved important for advocacy, dissemination of information and signifying trust between the target communities and research personnel.

So you go in and you have Village Health Workers, or another group called Nyaterros. Nyaterros is a Mandinka word referring to ‘friends of the eye’. The staff was local staff that the people already know. So during the campaign these are the same team members that lead the team.

(TR 1)

The districts whereby we treated everybody, we used the ophthalmic nurses, we used the scouts, the reporters, students from the schools. So those are the people we use during the treatment, during the distribution, because the job was very hectic.

(TR 2)

## Discussion

This qualitative study in The Gambia examined community barriers to malaria MDA trials by interviewing stakeholders and community members involved in a 1999 malaria study and a 2010 trachoma study.

First, interviews with community members revealed a preference for MDA during the dry season due to their farming obligations during the rainy season. Although a valid preference, this finding was contradicted by researchers and opinion leaders interviewed who felt that participation in the dry season would always be limited because of ceremonies and freedom to travel. The potential for maximal participation would be greater during the rain because of farming duties, but working demands and side effects of the medicine may be legitimate reasons to avoid MDA during this period. Soliciting preferences from the community in initial sensitization meetings could help solve this challenge, which is further explored in Theme 4, revolved around communication. Substantial evidence is lacking as to which season may be superior to employ MDA. However, MDA implemented just before or during the rainy season in The Gambia may have a significant impact given the highly seasonal nature of transmission in this area [21]. Several government officials suggested collaboration with Senegal, arguing the potential for increased participation through broad-scale implementation that caters to the natural movement to and from different communities.

Second, major barriers to compliance and adherence of MDA trials are a product of participants’ perceptions of the procedures, drug regimen, possible side effects, and eligibility for the trials. Although participation may initially be high, misconceptions and miscommunications typically arise over time and result in decreased participation. Villagers expressed a fear of blood sampling for malaria parasite detection, as it was drawn intravenously in the 1999 MDA trial. Given that malaria testing is currently done by rapid diagnostic test or microscopy in this setting (requiring only a finger prick blood sample), this fear is understandable and could be resolved by informing participants prior to the beginning of MDA that updated diagnostic methods no longer require intravenous blood samples. Further, villagers cited difficulty with the drug regimen due to the number of pills required. Explicit explanations of ACT dosage and possibility of side effects, with physical examples of tablet size and number, should be included in sensitization sessions. The number of pills and tablet size may vary across different ACT combinations, and the quantity of pills has been reported to deter compliance in sub-Saharan Africa [22]. Similar problems have been found in lymphatic filariasis MDA trials, with non-compliance resulting from fear of side-effects despite drugs being well-tolerated by participants [23,24].

Third, sensitization sessions were conveyed as the most essential component of a successful MDA trial. Not only do they provide overall information and allow community members to ask questions but, importantly, sensitization sessions also address cultural courtesies and establish a relationship between study populations and research staff. Although community acceptance of MDA trials has increased over the last few decades according to this study’s findings, sensitization still needs refining to ensure consistent and clear information is disseminated to study populations. For example, the attendance of key personnel, such as the Alkalo (village leaders), Imam (religious leader), and other leaders of groups (youth or women) may play an important role in community buy-in given their status as decision makers and leaders [25]. Additionally, identifying others in the area who may be diagnosing and treating malaria (such as private pharmacy workers) is important for comprehensive coverage. Such individuals may not be apparent to the organizers of MDA trials, but can be identified and included in sensitization sessions with the help of village leaders. Sensitization sessions should also be arranged well in advance of the MDA trial, as well as immediately before, during, and after it has commenced.

Media were not used as a promotional tool in the 1999 malaria MDA trial in The Gambia, but were a successful component of the trachoma campaign’s strategy according to key informants. Radio, television and billboards were important avenues for information dissemination and timing reminders, and increased the likelihood of reaching each community member. Information should be dispersed using a mixed-method approach, relying mainly on sensitization meetings with individual villages and supplemented by reminders and promotions through media. Villagers should be included during the planning of sensitization sessions to offer suggestions on how they should be run and who should be present.

Lastly, as with any top-down intervention, participants must communicate and collaborate cohesively. Communication was identified as the biggest challenge to an MDA trial, specifically the gap between fieldworkers (the individuals assisting researchers) and the villagers. This flow can be characterized bi-directionally, by information disseminated from fieldworkers to the community or by concerns articulated from the community to the fieldworkers and up to the research personnel. Lack of efficient and effective communication to villagers is likely due to inadequate fieldworker training. It is important that the fieldworker be able to articulate the future benefits and implications of the MDA trial, and to do so regularly given the constant testing and administering of drugs throughout the trial. Importantly, the information disseminated in either direction should be truthful, consistent and frequent, aiming to identify and resolve barriers to trials. Further, information should continue to be disseminated back to the communities even after the MDA trial has stopped. This will help foster the trust established between rural populations and the research community, and will help justify the villagers’ investment in the trial.

As accuracy and consistency of information were repeatedly reported as essential elements of sensitization, key informants also gave suggestions on how to best restructure an MDA to address this. Questions and concerns from villagers are apparent from the beginning of sensitization through the end of any such intervention. Therefore, consistent and readily available information is essential. A possible solution to several adherence issues could involve permanently posting a fieldworker in each participating village. Several fieldworkers and trachoma researchers relayed that there needs to be a person in the village that can be constantly relied upon by the general community for questions regarding the trial. This also might be an opportunity to take advantage of local health staff, like community health care workers, who would convey information to a supervising fieldworker. Additional training sessions would be required if including local health staff in an MDA trial. However, this training might be coupled with fieldworker training and therefore potentially alleviate barriers to community education and participation efforts. Involving traditional healers in MDA trials may also be important to demonstrate advocacy and enhance community participation [26]. Traditional healers are revered in rural communities in The Gambia, making them an important group to involve in sensitization sessions and the actual drug administration days [27]. It has been seen in other disease-specific interventions in The Gambia that advocacy and assistance from village healthcare workers can increase compliance and sustainability [28,29]. Therefore, future trials in The Gambia should specifically target village healthcare workers, include them in training, and solicit their help in information dissemination and during administrations.

### Limitations

This study has two distinct limitations, the first being that interviews may include biased views due to the inherent nature of snowball and purposive sampling. However, this limitation was recognized prior to sampling and the diversity of participants aimed to even out any possible bias. Secondly, information collected in this study is not only specific to The Gambia, but also specific to MDA trials for malaria. Therefore, results are likely not transferable to other settings, but may still be used to inform other MDA trials.

## Conclusions

Participation in malaria MDA trials may be maximized during the rainy season, when all villagers are working in the farms. However, villagers prefer not to have drugs administered during this time, so targeting the transition window between the rainy and dry seasons may be a successful compromise. Fears around blood sampling and medications given during the MDA trial should be addressed during sensitization meetings in advance of the actual trial administration. Importantly, accurate and frequent communication is essential, both to and from target populations. The use of local media as an adjunct to sensitization sessions may increase uptake within the community. In further malaria MDA trials, including a designated, trained fieldworker in each village during trial administration may increase compliance and reduce fears for participating residents.

## Abbreviations

ACT: Artemisinin-based combination therapy; MDA: Mass drug administration.

## Competing interests

The authors declare that they have no competing interests.

## Authors’ contributions

NJD conducted and authored the study. RDG and KAB supervised study design, data analysis, and manuscript preparation. UD’A and SJC assisted in coordinating work in the field. All authors read and approved the manuscript’s final draft.
